# Two Variants of the C-Reactive Protein Gene Are Associated with Risk of Pre-Eclampsia in an American Indian Population

**DOI:** 10.1371/journal.pone.0071231

**Published:** 2013-08-05

**Authors:** Lyle G. Best, Richa Saxena, Cindy M. Anderson, Michael R. Barnes, Hakon Hakonarson, Gilbert Falcon, Candelaria Martin, Berta Almoguera Castillo, Ananth Karumanchi, Kylie Keplin, Nichole Pearson, Felicia Lamb, Shellee Bercier, Brendan J. Keating

**Affiliations:** 1 Science Department, Turtle Mountain Community College, Belcourt, North Dakota, United States of America; 2 School of Medicine and Health Sciences, University of North Dakota, Grand Forks, North Dakota, United States of America; 3 Center for Human Genetics Research, Massachusetts General Hospital, Harvard Medical School, Boston, Massachusetts, United States of America; 4 College of Nursing, University of North Dakota, Grand Forks, North Dakota, United States of America; 5 William Harvey Research Institute, Barts and London School of Medicine and Dentistry, London, United Kingdom; 6 Center for Applied Genomics, The Children's Hospital of Philadelphia, Philadelphia, Pennsylvania, United States of America; 7 Center for Vascular Biology Research, Beth Israel Deaconess Medical Center, Harvard Medical School, Boston, Massachusetts, United States of America; Yale School of Public Health, United States of America

## Abstract

**Background:**

The etiology of pre-eclampsia (PE) is unknown; but it is accepted that normal pregnancy represents a distinctive challenge to the maternal immune system. C-reactive protein is a prominent component of the innate immune system; and we previously reported an association between PE and the *CRP* polymorphism, rs1205. Our aim was to explore the effects of additional *CRP* variants. The IBC (Cardiochip) genotyping microarray focuses on candidate genes and pathways related to the pathophysiology of cardiovascular disease.

**Methods:**

This study recruited 140 cases of PE and 270 matched controls, of which 95 cases met criteria as severe PE, from an American Indian community. IBC array genotypes from 10 suitable *CRP* SNPs were analyzed. A replication sample of 178 cases and 427 controls of European ancestry was also genotyped.

**Results:**

A nominally significant difference (p value <0.05) was seen in the distribution of discordant matched pairs for rs3093068; and Bonferroni corrected differences (*P*<0.005) were seen for rs876538, rs2794521, and rs3091244. Univariate conditional logistic regression odds ratios (OR) were nominally significant for rs3093068 and rs876538 models only. Multivariate logistic models with adjustment for mother's age, nulliparity and BMI attenuated the effect (OR 1.58, *P* = 0.066, 95% CI 0.97–2.58) for rs876538 and (OR 2.59, *P* = 0.050, 95% CI 1.00–6.68) for rs3093068. An additive risk score of the above two risk genotypes shows a multivariate adjusted OR of 2.04 (*P* = 0.013, 95% CI 1.16–3.56). The replication sample also demonstrated significant association between PE and the rs876538 allele (OR = 1.55, *P* = 0.01, 95% CI 2.16–1.10). We also show putative functionality for the rs876538 and rs3093068 *CRP* variants.

**Conclusion:**

The *CRP* variants, rs876538 and rs3093068, previously associated with other cardiovascular disease phenotypes, show suggestive association with PE in this American Indian population, further supporting a possible role for *CRP* in PE.

## Introduction

Pre-eclampsia (PE) is a pathologic condition of pregnancy characterized by the onset of hypertension and proteinuria after 20 weeks of gestation.[1], [2] It has been called a “disease of theories”[3], [4] due to multiple putative etiologies and risk factors; but placental ischemia seems to play a central role in the pathogenesis, which involves an imbalance of circulating angiogenic factors and endothelial dysfunction.[5], [6] The underlying cause of this ischemia remains unknown; however pregnancy has long been recognized as a distinctive challenge to the maternal immune system. Maladaptive responses to this immune challenge are frequently postulated as primary initiators of the multiple subsequent pathways leading to PE. [7], [8]

Although there is significant phenotypic heterogeneity across the spectrum of PE, a number of studies have shown a 2–5 fold increase in risk amongst first-degree relatives of women with PE.[9]–[12] Twin studies estimate the heritable component of PE to be >50% with high concordance for PE amongst mono-zygotic twins (OR 33.6 [95% CI: 7.8–145]).[13] Cnattingius et al found that 35% of the variance in PE was attributable to maternal genetic effects, 20% to fetal genetic effects (with similar contribution of maternal and paternal genetic effects), 13% to the ‘couple effect, and 32% to unmeasured factors.[14] Autosomal dominant inheritance with 50% penetrance or multifactorial causation, matrilineal and mitochondrial inheritance have also been proposed as a plausible models of inheritance.[11], [15] A variety of genetic approaches have been applied to the study of PE including linkage analysis and candidate gene studies. Whole-genome linkage screens revealed at least eight chromosomal regions affecting PE transmission.[16]–[18] Delineating the 2q22 linkage peak revealed variants in the ACVR2A locus to be associated with PE in a Norwegian PE population.[19] Johnson and colleagues have also resolved a linkage signal in Chromosome 5q, demonstrating association of *ARTS-1* in one of the few large well powered PE studies.[20] Founds et al. (2009) reported evidence in early pregnancy of dysregulated genes associated with immune function in chorionic villus sampling (CVS) tissues.[21] Most PE candidate gene association studies have focused on genes relating to: (a) renin–angiotensin system; (b) coagulation factors; (c) oxidative stress pathways; (d) dyslipidemia; and (e) immunoregulatory components, in particular within the HLA region. The incidence of PE is frequently reported to be approximately 5–8% of pregnancies.[22] in most populations; but the only systematic assessment in an American Indian population found a prevalence of 9.1% among pregnancies in the Navajo Nation.[23] Significantly higher rates of hypertensive disorders of pregnancy in women of African American versus European ancestry have been observed.[24]

C-reactive protein (CRP) is a prominent component of the innate immune system[25]; and has been employed as a non-specific measure of inflammatory status in epidemiologic studies of cardiovascular disease (CVD).[26] Although the expression of CRP in relation to the etiology and clinical severity of both hypertension and PE has been examined extensively [27]–[30]; there has been a lack of information on the possible association between genetic variants of *CRP* and PE.

Previously we demonstrated an association between rs1205 in *CRP* and severe PE in an American Indian cohort;[31] and the present study was undertaken to determine if other genetic variants of *CRP* captured on the IBC array are correlated with risk or severity of PE within this higher risk population.

## Methods

### Study recruitments and Ethical Approval

#### Turtle Mountain Community College (TMCC)

Approval was obtained from the Aberdeen Area IHS and University of North Dakota Institutional Review Boards (IRBs) and the tribal government. Written informed consent was obtained from each participant.

#### Boston study

The Institutional Review Board (IRB) of record for Massachusetts General Hospital and Brigham and Women's Hospital is Partners Human Research Committee; and for Beth Israel Deaconess Medical Center it is the Committee on Clinical Investigations. With IRB approval, the billing records of patients delivered at Massachusetts General Hospital and Brigham and Women's Hospital from 1995–2011 were queried for ICD9 codes suggestive of PE. Whenever a PE patient returned to a Partner's Hospital, blood samples at their point of discard (after completion of all clinical diagnostic testing) were collected for genetic analysis. Cases were validated for PE and its severity by electronic medical record/chart review in a 30 day time-limited link to protected health information. After links to protected health information were deleted, extracted DNAs of accessioned samples were released to the study staff in a de-identified manner for further investigation. Because the investigators do not interact with any individuals for the ascertainment of data or samples, informed consent was not obtained: the Partners Human Research Committee elected to waive the requirement to seek informed consent (as detailed by 45 CFR 46.116). The participants from Beth Israel Deaconess Medical Center provided written, informed consent.

#### Philadelphia study

The Children's Hospital of Philadelphia (CHOP) IRB approved DNA collection from 73 mothers with PE and 353 control normotensive mothers from 2009–2012 at the Center for Applied Genomics (CAG) at CHOP through the Study of the Genetic Causes of Complex Pediatric Disorders (GCPD). Mothers of recruited children were asked between 2009–2012 if they had ever had PE during their pregnancy and gave written informed consent and HIPAA authorization to allow access to their medical records.

Recruitment for the TMCC case and matched control study has been ongoing from 8/04 to 7/12. The federally funded Indian Health Service (IHS), through the hospital and clinic located in Belcourt, North Dakota, is the primary health care provider for eligible tribal members of the Turtle Mountain Band of Chippewa. Most potential cases (∼80%) were identified by automated query of an electronic medical record database (the Resource, Patient, Management System [RPMS]) at this facility, using a relevant group of ICD9 codes, designed to be inclusive. Additional potential cases (∼20%) were “self-identified” among family members and acquaintances of cases and during the recruiting of controls.

The medical records of all potential cases were abstracted for 78 clinically relevant factors, including the highest of up to 3 blood pressure (BP) measures between 20 weeks of gestation and 30 days postpartum and the highest of up to 2 measures of proteinuria in the same period. Cases were verified as meeting diagnostic criteria for PE if at least 2 of the following were identified:

1. At least 3 BP values above either 140 mmHg systolic or 90 mmHg diastolic; and absence of a diagnosis of, or treatment for hypertension (during the year prior to conception and the first 20 weeks of gestation).
2. Proteinuria as indicated by a 24 hour excretion of >300 mg, or at least two +1 dipstick measurements in the absence of prior proteinuria.
3. A diagnosis of PE, eclampsia, or the hemolysis, elevated liver enzymes, low platelet (HELLP) syndrome by an attending physician after 20 weeks of gestation.

These criteria were chosen to be compatible with the NHLBI Working Group on Research on Hypertension in Pregnancy (Working Group) definition [32] and to also consider the clinical judgment of the attending physician. Since the present study uses a retrospective, case/control design, it was not possible to mandate specific measures at defined intervals. The cases and controls were ascertained from records spanning more than 15 years and including over 6 facilities. It should be noted that the present study criteria include repeat measures, which while recommended and encouraged, are not strictly required according to the Working Group[32] or the American Society of Hypertension (ASH).[33] These advisory groups have also stressed the need for careful attention to subjective symptoms and less common signs. For all of these reasons, it seems justified to give partial weight to the attending physician's diagnosis in addition to the more easily quantified measures.

Controls were ascertained by contact of the first individual to deliver before and after the index case. If a potential control declined participation, the woman delivering during the next prior or subsequent day was contacted; and this was continued until two controls were recruited, one before and one after the index case. This method of ascertaining controls was chosen as a convenient means of randomization and to control for possible seasonal influences on PE.[34] Some participants had been recruited from the prenatal clinic at this facility as part of an anticipated longitudinal cohort, which became impractical. After criteria for control status was confirmed, they were matched by date of delivery as closely as possible to previously identified cases, up to a maximum of 3 months from the index case. These additional controls involved 65 (24%) of the pairs. Of these alternatively recruited controls, 50 (77%) were matched to within 30 days of their case and only 4 differed by more than 60 days (1 control a maximum of 72 days) from their case. As a group these additional controls differed from their cases by an average of 18.57 days, compared with 9.39 days (p<0.001) for the originally matched pairs. The available medical records of all controls were abstracted in the same way as cases; and matching of controls was entirely blinded to genotype. It was verified that these individuals did not meet criteria for PE. Birth certificate data were also obtained for case/control participants to more uniformly ascertain data on such factors as smoking, alcohol intake and educational attainment.

Replication analysis was conducted on two groups assembled for a larger collaborative study, comprised of:

1. The Boston area Pre-eclampsia Genetics Collection (three Harvard affiliated hospitals).
  Samples meeting ACOG 2002 criteria were included in the study (88 cases). Blood from 17 additional cases based on physician diagnosis and 74 normal term controls were collected from patients at the Beth Israel Deaconess Medical Center.
2. Children's Hospital of Philadelphia Pre-eclampsia Genetics Collection.

The definition of PE employed is defined as onset hypertension after 20 weeks of gestation with systolic BP≥140 or diastolic BP≥90 on two occasions at least 6 hours apart in the presence of proteinuria of 300 mg/dL on a 24 hour collection or at least +1 on a dipstick.

For the American Indian population, prior to January of 2009, template DNA was provided by capillary blood samples collected on “FTA Classic Cards” (Whatman Inc) paper. Three 1.2 mm diameter “pellets” were punched from the cards and processed according to the manufacturer's recommendations. Recently template DNA has been collected and processed using salivary samples and the Oragene (DNA Genotek Inc) system; and the majority of those with capillary samples were re-consented and an additional salivary sample obtained. For genotyping of the replication cohort, DNA was extracted from blood or saliva using standard alkaline lysis methodologies and stored at −20°C.

Genotyping was accomplished primarily (319 of 410 (78%) total samples) by microarray analysis on the ITMAT/Broad/CARe (IBCv1) at The Center for Applied Genomics at CHOP. This genotyping microarray selected >49,000 SNPs related to ∼2,000 genes and was guided by genome-wide association study data, pathway based approaches and comprehensive literature searches to variants with known relevance to cardiovascular, metabolic and inflammatory pathology.[35] Quality control standards were monitored with the mean call rate above 98% for all SNPs on the microarray and less than 4% of samples had a SNP call rate below 95%. The call rates for rs876538 and rs3093068 were 100% and 95.3% respectively. Twenty four samples were genotyped for the rs876538 variant with both the IBC array and TaqMan (Life Technologies) with no discordant results observed.

Results of the microarray genotyping will be available to qualified investigators with assurances that 1) no attempts will be made to identify individuals, 2) goals of the analysis are within the scope of the consent and 3) the results will not be used for commercial purposes.

The remainder of the samples without salivary DNA were genotyped using pre-designed TaqMan genotyping assays and protocols were implemented for SNPs on a real-time, Mini-Opticon (Bio-Rad Laboratories Inc), four color thermocycler. Controls were identified for at least two of the three possible genotypes (and “blank” controls) for each SNP were included with each analysis. Control material of consistent genotype (replicated a minimum of ten times) for each genotype was run with each set of samples. In the case of rs876538, HapMap genotypes from eight Coriell Institute reference samples were confirmed in our laboratory.

The replication genotyping for this study, European ancestry samples from Boston and Pennsylvania (n = 605), was also conducted on the IBC array with principal components analysis performed using standard Eigenstrat pipelines.[36] Quality control criteria included filtering of samples with <90% call rate (0 removed) and SNPs with <90% call rate, minor allele frequency <1% or departure from Hardy Weinberg equilibrium with *P*<10^−6^. The rs1205 and rs876538 variants passed quality control but rs3093068 failed within this replication sample, therefore we used the proxy rs1206785 (r^2^ = 1 with rs3093068 in 1000 Genomes project [1KGP] CEU population).[37] Overall, 178 cases and 427 controls were available for association analysis within the replication population.

The primary analysis of the American Indian samples chose all 24 *CRP* SNPs available on the IBCv1 array. Of these, eight were observed to be monomorphic in this population and another five were excluded for low prevalence. Another SNP exhibited a call rate of 60% and was considered unreliable for analysis, leaving ten SNPs remaining for analyses.

Statistical analysis was primarily carried out using SPSS version 10.1.0 software, with Egret version 2.0.31 used for the logistic regression analysis. Descriptive statistics report mean (+/− SD) for continuous variables and proportions with 95% CI for discrete variables. HWE analysis was assessed using standard chi-square methods. Due to the matched nature of case/control pairs, McNemar chi square analysis is required for genotype comparisons; and relies on evaluation of the two opposing sets of discordant genotypes. The null hypothesis holds that there should be equal numbers of discordant pairs where the case has genotype A and the matched control has genotype B; compared with the opposite situation, where the case has genotype B and the control, genotype A. The genotype risk score for McNemar analysis considered the presence of either identified risk genotype, rs3093068 (G allele dominant) or rs876538 (C allele recessive), among pairs with both alleles available. The risk score for logistic regression analysis used a more powerful, ordinal variable, where both risk genotypes  = 2, either  = 1, and none  = 0. This ordinal risk score could not be used for the McNemar paired analysis because the available software will not support it and many statistical texts consider this analysis “beyond their scope”. Conditional logistic regression was used to explore the multivariate association of genotype and other variables with risk of PE. Statistical significance was set at *P*≤0.05, and study-wide statistical significance at *P* = 0.005 after Bonferroni correction. Principal component analysis was conducted using ancestry informative SNPs from the IBCv1 microarray.

The replication analysis was performed using logistic regression with SNPs coded in an additive genetic model, and with adjustment for study site and 10 principal components computed as described above to correct for population structure.

### Genomic characterisation and SNP functional analysis

The extended locus around each associated SNP was defined by identification of all SNPs showing r^2^ LD>0.5. LD was defined in the three HapMap population groups using the HaploReg tool which includes LD data derived from Phase I 1000 genomes project. Population groups were CEU (Utah Residents (CEPH) with Northern and Western European ancestry), ASN (Han Chinese in Bejing, China; JPT Japanese in Tokyo, Japan; CHS Southern Han Chinese; CDX Chinese Dai in Xishuangbanna, China; KHV Kinh in Ho Chi Minh City, Vietnam) and AMR (MXL Mexican Ancestry from Los Angeles USA; PUR Puerto Ricans from Puerto Rico; CLM Colombians from Medellin, Colombia; PEL Peruvians from Lima, Peru). The full genomic context of the CRP locus defined by this process is shown in Figures S1 and S2 and the SNPs identified in this region are shown in Table S1. The putative functional impact of associated SNP signals were investigated using a combination of HaploReg [38] and Regulomedb[39], which both draw on comprehensive data from the Encyclopedia of DNA Elements (ENCODE), including published eQTL studies.[40] All SNPs identified in LD with the associated variants were visualized in the UCSC human genome browser.

## Results

Among the 140 cases of the discovery sample, 52 (37%) utilized the clinical diagnosis as one of only two qualifying criteria. Of these 52, all but 11 (8%) would have met either the minimal standards for BP or proteinuria of the Working Group or ASH; and all of those 11 met at least the more stringent BP/proteinuria standard of the current study, in addition to a clinical diagnosis of PE. Five of these 52 cases also had objective signs of end organ involvement as defined below and three had seizures.

At least one of the American College of Obstetricians and Gynecologists' (ACOG) defining criteria [41] for severe PE was met by 95 (68%) cases. These criteria require two BP measures over 160 systolic or 110 diastolic, separated by at least 6 hours; and proteinuria exceeding 5 grams in 24 hours or 3+ by dipstick on two occasions. Of those with severe PE, 84 (88%) had at least two BP measurements over 160 systolic or 110 diastolic, 22 (23%) met the 3+ proteinuria by dipstick or over 5 gm per 24 hour criteria, and 16 (17%) individuals met both criteria. In the context of a hypertensive pregnancy, an additional 5 cases had a combination or any one of: platelets less than 100,000/mm^3^, creatinine greater than 1.3 mg/dl, liver transaminases over two times the upper limit of normal for the hospital laboratory, or experienced a seizure during parturition, also meeting criteria for severe PE.

Table 1 summarizes the SNPs tested, population prevalence and consistency with HWE among cases and controls combined. None of the SNPs tested showed significant deviation from HWE. As the design of the IBC array captured specific CVD-related SNPs from the previous literature, as well as tagged all common variation across *CRP*, all of the SNPs (except rs3093070 – which had design/synthesis issues) have been captured.

**Table 1:** Characteristics of SNPs studied and study population prevalences.

dbSNP ID	Physical position (bp)	Alleles, functional effect	Minor allele frequency	95% CI	Hardy-Weinberg (p value)
rs12068753	159692537	T/A, 5′ UTR	A = 5.8%	4.1–7.6	0.251
rs1341665	159691559	G/A, 5′ UTR	A = 44.9%	41.0–48.7	0.443
rs12084589	159688441	C/A, 5′ UTR	A = 5.7%	4.0–7.4	0.263
rs3093059	159685136	T/C, 5′ UTR	C = 5.8%	4.1–7.6	0.251
rs2794521	159685096	T/C, 5′ UTR	C = 21.7%	18.5–24.9	0.101
rs3091244	159684665	C/T, 5′ UTR	T = 30.2%	26.7–33.6	0.698
rs1800947	159683438	C/G, coding, synon	G = 4.0%	2.5–5.5	0.517
rs3093068	159681364	C/G, 3′ UTR	G = 5.9%	4.1–7.8	0.252
rs3093070	159680817	A/C, 3′ UTR	C = 0.8%	0.1–1.5	0.885
rs876538	159675717	C/T, 3′ UTR	T = 18.4%	15.8–20.9	0.206
Boston and Philadelphia replication cohort					
dbSNP ID		alleles,functional effect	Minor allele frequency	95% CI	Hardy-Weinberg (p value)
rs 12068753 proxy for rs3093068	159692537	T/A, 5′ UTR	A = 7%	5.2–8.8	1.00
rs876538	159675717	C/T, 3′ UTR	T = 21%	18.2–23.8	0.14
rs1205	159682233	C/T, 3′ UTR	T = 35%	31.7–38.3	0.36

Pertinent characteristics of all paired cases and controls are summarized in Table 2. Of the ten SNPs studied, rs2794521 and rs876538 revealed nominally significant association (*P*<0.05), whereas rs3091244 and rs3093068 showed marginally significant differences between cases and controls. When comparing allelic frequency of severe PE cases and controls, none of the SNPs retained significance. Significant differences between all cases and controls were noted for nulliparity, weeks of gestation at delivery, body mass index (BMI), gestational diabetes, weight at first prenatal visit, and both systolic and diastolic blood pressure. Differences in near term delivery, birth weight of infants and BPs were not included in further models and felt likely to be consequences of PE or the applied diagnostic criteria, rather than etiologic. Principal component analysis of the ten vectors showed no significant difference between cases and controls.

**Table 2:** Characteristics of matched cases and controls.

Characteristic	Cases	Controls	p value
Number (N)	140	270	N/A
rs2794521, C allele freq	41/201* = 16.9%	92/254 = 26.6%	0.008**
rs3091244, T allele freq	87/169 = 34.0%	101/281 = 26.4%	0.050
rs3093068, G allele freq	20/246 = 8.1%	16/366 = 4.4%	0.078
rs876538, T allele freq	40/280 = 14.3%	119/440 = 27%	0.010
Age, mean years (SD)	23.88 (6.4)	23.74 (5.40)	0.768***
Parity (N,% nulliparous)	94/140 (67.1%)	114/270 (42.2%)	<0.001****
Weight at first prenatal	181.7 (46.82)	161.6 (41.35)	<0.001***
Body-Mass index (BMI)	30.92 (7.39)	27.42 (6.88)	<0.001***
Gestational diabetes, N (% yes)	15/122 (12.3%)	9/199 (4.8%)	0.031****
Weeks of gestation at delivery	36.67 (3.96)	39.52 (1.82)	<0.001***
Birth weight of infant, grams	3013 (967)	3431 (623)	<0.001***
Mother's educational attainment (years of education)	11.95 (1.85)	12.15 (2.44)	0.405***
Maternal smoking, N (% yes)	38/95 (40.0%)	76/160 (47.5%)	0.543****
Maternal smoking (Mean cigarettes smoked)	6.75 (18.03)	10.51 (23.20)	0.047***
Mean systolic blood pressure	163.8 (18.3)	132.9 (12.9)	<0.001***
Mean diastolic blood pressure	97.6 (10.1)	78.6 (8.4)	<0.001***

*41/201 indicates 41 minor alleles (T in this case) and 201 major alleles for a total of 242.

**Differences in allele frequency evaluated with Chi square test.

***Differences between means evaluated with paired t test.

****Differences between discrete variables evaluated with McNemar's Chi square test.

Table 3 shows the genotypic results of paired cases and controls. The number of pairs with discordant genotype and the total number of pairs analyzed is shown for each of the 10 SNPs tested in both the total cohort and in the severe PE subset. McNemar chi-square analysis of pair wise comparisons (major allele dominant, minor allele dominant) demonstrates nominally significant associations for all cases of rs3093068 (G dominant), and Bonferroni adjusted, significant associations for rs2794521 (C dominant), rs3091244 (C dominant), and rs876538 (C recessive) and the dichotomous risk score including those with either genotype, with chi square, 1 df, values ranging from 8.22 to 16.83 (*P* = 4×10^−5^). For analyses considering only severe cases, the only comparisons that retained significance were rs876538 (C recessive), nominal; and the dichotomous risk score (*P*<0.0001), significant after Bonferroni adjustment. The two SNPs of primary interest are modestly correlated with each other (r^2^ = 0.014, D′ = 1.0) and with rs1205 (r^2^ = 0.03, D′ = 0.84 for rs3093068 and r^2^ = 0.16, D′ = 0.97 for rs876538).

**Table 3:** Genotypes associated with case/control (matched-pair) status.

		Discord pairs*				Discord pairs			
dbSNP	allele	Maj Dom	Alter	p value	allele	Min Dom	alter	p value	total pairs
rs12068753	T	28	15	0.07	A	0	0	NA	177
rs1341665	G	33	33	0.90	A	24	21	0.77	152
rs12084589	C	27	15	0.09	A	0	0	NA	176
rs3093059	T	0	0	NA	C	28	15	0.07	177
rs2794521	T	7	5	0.77	C	25	51	0.004	153
rs3091244	C	59	29	0.002	T	13	24	0.10	177
rs1800947	C	1	0	1.00	G	16	8	0.15	172
rs3093068	C	0	0	NA	G	29	13	0.021	163
rs3093070	A	0	0	NA	C	3	3	0.68	145
rs876538	C	8	6	0.789	T	40	82	<0.001	270
risk score**	yes	57	20	<0.001					
Severe Cases and Controls									
rs12068753	T	19	11	0.201	A	0	0	NA	117
rs1341665	G	21	18	0.749	A	14	13	1.0	95
rs12084589	C	18	11	0.265	A	0	0	NA	116
rs3093059	T	0	0	NA	C	19	11	0.201	117
rs2794521	T	5	5	0.752	C	19	35	0.041	106
rs3091244	C	34	23	0.185	T	11	17	0.348	117
rs1800947	C	0	0	NA	G	9	5	0.423	115
rs3093068	C	0	0	NA	G	22	9	0.031	108
rs3093070	A	0	0	NA	C	3	2	0.149	102
rs876538	C	7	6	1.0	T	31	58	0.006	186
risk score	yes	41	12	0.0001					108

*“Discord pairs” indicates the number of case/control pairs that are discordant for a particular genotype “Maj Dom”: where the major allele is dominant for the case. “Alter” indicates the alternate situation where the control genotype is dominant for the major allele. Similarly “Min Dom” indicates discordant pairs where the case genotype is dominant for the minor allele. “Total pairs” shows the total number of matched pairs, including both concordant and discordant pairs. “NA” refers to “not applicable”.

**Risk score indicates the risk genotype from either rs3093068 (G allele dom) or rs876538 (C allele recess) is present, among pairs with both alleles available.

Univariate conditional logistic regression results are shown in Table 4 and confirm frequently reported associations between nulliparous status, maternal obesity and infant birth weight. [33], [34] Gestational diabetes also showed a significant association with PE in univariate analysis; but lacked significance (*P* = 0.117) when included in a multivariate model with age at delivery, nulliparity and BMI. The SNP rs876538 showed nominal significance in analysis of all cases; and the additive risk score was nearly significant with a Bonferroni adjusted p value of 0.009, whereas rs2794521, rs3091244, and rs3093068, demonstrated only marginal significance (*P* = 0.068, *p* = 0.078, *P* = 0.063 respectively) in univariate analysis. Models utilizing multivariate conditional logistic regression (Table 5) continued to show robust, independent effects of nulliparity and obesity. Since age is clearly related to nulliparity, inclusion in multivariate models was deemed necessary, although models without showed very similar results. Multivariate models of the individual SNP genotypes were attenuated to marginal significance for rs2794521 and rs876538 (both *P* = 0.066). The additive risk score showed nominally significant association with PE among all cases and controls (*P* = 0.016).

**Table 4:** Univariate, conditional logistic regression analysis of factors associated with pre-eclampsia.

Characteristic	N pairs	Model	OR	p Value
*CRP*, rs3093068, (G allele), All pre-eclampsia cases	163	Additive	1.973	0.063
		Dominant	1.973	0.063
		Recessive	N.A.	
*CRP*, rs3093068, (G allele), Severe pre-eclampsia	108	Additive	2.368	0.041
		Dominant	2.368	0.041
		Recessive	N.A.	
*CRP*, rs876538, (C allele), All pre-eclampsia cases	270	Additive	1.502	0.045
		Dominant	0.803	0.746
		Recessive	1.611	0.033
*CRP*, rs876538, (C allele), Severe pre-eclampsia	186	Additive	1.361	0.184
		Dominant	0.890	0.866
		Recessive	1.477	0.141
rs3093068,(dom) and rs876538 (recess) risk score, both = 2, either = 1, neither = 0	163	risk score	1.688	0.009
rs3093068,(dom) and rs876538 (recess) above with severe PE	108	risk score	1.820	0.012
Age at delivery (per year)	270		0.996	0.843
Nulliparity (yes)	270		3.026	0.001
Gestation at first prenatal visit (per week from LMP)	178		0.978	0.190
Weight at first prenatal (per pound)	265		1.008	0.001
Body-Mass index (per unit Kg/meter^2^)	265		1.054	0.001
Birth weight of infant (per gram)	147		0.999	0.001
Mother's educational attainment (per year)	144		0.948	0.407
Maternal smoking (mean cigarettes smoked per day)	144		0.985	0.091
Gestational diabetes in current pregnancy (yes)	189		2.369	0.034

Boston and Philadelphia replication cohort, univariate logistic regression				
Characteristic	Cases/Controls	Model	OR	p Value
CRP, rs 12068753 (A allele) for rs3093068	178/424	Additive	0.940	0.810
*CRP*, rs876538 (C allele)	178/423	Additive	1.547	0.011
*CRP*, rs1205 (T allele)	173/410	Additive	1.037	0.787

**Table 5:** Multivariate, conditional logistic regression analysis of factors associated with pre-eclampsia and severe pre-eclampsia.

	Pre-eclampsia		Severe Pre-eclampsia	
*MODEL 1, all of following* *				
	OR	P value	OR	P value
Age at delivery	1.053	0.076	1.052	0.166
Nulliparous	5.600	0.001	4.170	0.001
BMI	1.061	0.002	1.059	0.001
Gestational diabetes	1.684	0.278	2.241	0.166
*MODEL 2, Age, nulliparity,and BMI,plus each of the following individually*:**				
*CRP*, rs3093068, (G allele additive)	1.904	0.124	2.587	0.050
*CRP*. rs3093068, (G allele recessive)	N.A.		N.A.	
*CRP*, rs3093068, (G allele dominant)	1.904	0.124	2.587	0.050
*CRP*, rs876538, (C allele additive)	1.524	0.060	1.319	0.271
*CRP*, rs876538, (C allele recessive)	1.583	0.066	1.380	0.267
*CRP*, rs876538, (C allele dominant)	0.532	0.404	0.699	0.637
rs3093068,(G dom) and rs876538 (C recess) additive risk score, both = 2, either = 1, neither = 0	1.779	0.016	2.035	0.013

*Covariates showing univariate significance, plus age, which interacts with nulliparity.

Univariate analysis of the 95 cases meeting the definition of severe PE and their 186 matched controls did not change any of the previously mentioned univariate relationships with clinical factors; and now showed nominally significant association with rs3093068 (OR 2.37, *P* = 0.04, 95%CI 1.03–5.42); but not with any of the other single SNPs genotypes. The additive risk score demonstrated nominal significance (OR 1.82, *P* = 0.012, 95%CI 1.14–2.91). Multivariate conditional logistic regression results showed marginal, independent association for rs3093068, p = 0.050, but lack of significance for the other individual SNPs. The additive risk score attained nominal significance, with an OR = 2.03, p = 0.013, and 95% CI 1.16–3.56.

Univariate replication results in the Boston and Philadelphia sample of European ancestry are shown at the bottom of Table 4. We observed significant replication of SNP rs876538 T with PE (OR (95% CI) 0.65 (0.46–0.90) p = 0.0109 for the minor T allele). T allele frequency was similar to the frequency observed in American Indians. No association of SNP rs1205 or rs12068753 was observed with risk of PE.

Review of LD with the associated SNPs rs876538 and rs3093068 identified 55, 32 and 39 variants showing LD (r^2^>0.5), in HapMap CEU, ASN and AMR populations respectively. Review of Haploreg and Regulomedb results showed multiple variants are predicted to be located in regulatory active regions (see Figures S1, S2 and Table S1). Review of the associated variants in the UCSC genome browser, identified a liver EST sequence BG616599 and evidence of transcription by RNA-seq exclusively in the liver, in the region containing rs3093068. This suggests that rs3093068 may possibly be located in the extended 3′ untranslated region of the CRP transcript. This presents the possibility that the variant could alter CRP mRNA stability of miRNA binding to the CRP transcript. Another published study by Choi et al. (2007) may also be highly relevant.[42] They investigated the mechanism of TNF-induced CRP expression and found that β-catenin enhanced the expression of CRP mRNA in concert with the p50 subunit of NF-κB, subsequently identifying a binding site for the β-catenin/TCF-4 complex in the downstream region of *CRP* by chromosome conformation capture. The SNP rs876538 is within 30 bp of one of the TCF-4 binding sites identified by Choi et al. and therefore may have an impact on CRP expression.

## Discussion

Among many proposed theories related to the etiology of PE, dysfunctional immune responses have played prominent roles.[43] C-reactive protein is an important component of the innate immune response and a large body of evidence has been developed showing correlation between CRP levels and other indicators of inflammatory states [29], [44], as well as associations with PE[45], [46] and multiple cardiovascular phenotypes with possible relevance to PE.[47] We previously identified an association between rs1205 in *CRP* with severe PE in this cohort of American Indian women.[31] These findings prompted the extension of our investigation into additional variants in *CRP* for association with PE in this higher risk risk population. Of the ten IBC array SNPs with sufficient prevalence to allow analysis of their relation with PE, there were two (rs3093068 and rs876538) that were PE-associated at nominal significance using both McNemar chi-square and logistic analysis; and three (rs876538, rs2794521, rs3091244) attained Bonferroni adjusted significance in the chi square, paired analysis. An additive risk score using these two alleles showed Bonferroni adjusted significance in both chi square (all and severe cases) and significant *P* values of 0.009 and 0.01 in univariate and multivariate logistic analysis respectively. While these variants in the *CRP* gene have been investigated for their association with other inflammatory conditions, to our knowledge, this is the first report of association between these two SNPs (in addition to rs1205) and PE. The replication of these findings in a population of European ancestry increases our confidence in these results.

The extent and specifics of genetic influences on serum CRP levels remains unclear; but a number of studies have identified important associations with various SNPs, [48]–[50] including those identified in this study. The 1KGP has reported a rs3093068 minor allele frequency of 6.7% among 120 European chromosomes [51], consistent with the current study. Carriers of this minor allele have increased levels of CRP, most notably in a subcohort of Asian/Pacific Islanders, suggesting the existence of ethnic variations in the relationship between rs3093068 and CRP concentrations.[52] In terms of association with clinical phenotypes, a haplotype tagged by rs3093068, rs1205 and another SNP was associated with risk of myocardial infarction in a large prospective study;[53] whereas a small, case/control study failed to find a relationship between neovascular age-related macular degeneration and rs3093068. [54] The lack of association with this SNP in our replication population may be due to the use of a proxy SNP that was in complete linkage disequilibrium in the European population; but less so in both our discovery cohort (r^2^ = 0.792) and the Asian population referenced above (r^2^ = 0.764 in 1000 G HapMap Asians (CHB/JPT)).[37] Another possibility is that rs3093068 is truly not associated with PE in the European replication population, due to the different genetic background.

The other SNP with significant findings of association in the present study, rs876538, has a prevalence very similar to the 22.1% reported for Europeans.[55] Increases in CRP levels in a predominantly older postoperative Caucasian male sample were not associated with rs876538, but were associated positively with rs3091244 and negatively with rs1800947. [56] The rs3091244 SNP did show association in our study during McNemar chi square analysis; but this was not supported after multivariate adjustment. The rs1800947 SNP failed to show any significant association in this study. Another SNP (rs2808630) in linkage disequilibrium with rs876538 (r^2^ = 0.63, d′ = 1) affects CRP expression.[57] However, rs876538 was associated, in an Australian population, with improved treatment response in neovascular age-related macular degeneration. [58]

In evaluating the findings of the present analysis and comparison with other association studies of CRP levels and PE, consideration must be given to genetic background effects such as: 1) differences in linkage disequilibrium between populations, causing discordant results due to the presence or absence of linkage to an unknown causative SNP, 2) differences in power to detect association due to different population prevalence for the SNP of interest and 3) differential effects of the SNP of interest based on interaction between the SNP of interest and an unknown genetic variant with population-specific effects. Furthermore, environmental factors can vary substantially between populations and overwhelm the genetic signal, resulting in a loss of power, or differential SNP-environment effects may influence the comparison between populations. The adjustment for age, BMI and gender in the present primary study may increase the likelihood that the identified SNPs are influencing the immune response, provided the modeled adjustment is accurate (i.e., first or second order, or bimodal distributions may complicate the adjustment).

Although the heritability of CRP in another American Indian population was found to be approximately 40% [59], there are clearly a number of environmental factors impacting CRP levels. The differential genetic and environmental effects on CRP serum levels and clinical phenotypes are complex and remain unclear. Clinical correlates including age, gender, BMI and BP were more strongly associated with CRP levels than *CRP* SNPs in a large cohort study of older Americans. Although rs876538 was included in the analysis, it did not retain significance after multivariate adjustment.[60]

The association between PE and inflammation has suggested CRP levels as an important marker of this disease. [45], [53], [61], [62] Although there have been contrary reports,[63] including evidence that the association may be seen only among lean individuals,[64] a systematic review found suggestive evidence for a prospective association between PE and elevated CRP which was further modified when BMI was also increased.[46]

None of these purported associations, either cross-sectional or prospective, prove that CRP is in the direct causal chain for PE, it may be simply a marker of inflammation initiated by current or still latent primary factors causing PE. The results of genetic association studies such as this do contribute to our understanding, in so far as the genetic variants are obviously present prior to the establishment of any disease state. None the less, CRP variants could still be either simple biomarkers, indicating a genetic heightened sensitivity to the primary cause(s) of PE; or they could be involved in pathogenesis as a modifier of response to an actual cause of the condition. Two publications make a similar observation when they suggest that CRP may be an intermediary between BMI and hypertension.[61], [65] Our previous findings demonstrated an association of PE with rs1205 genotypes linked to increased CRP levels, and are consistent with these studies. The present study reveals evidence of associations between rs3093068 and rs876538 by a number of statistical measures, and while some measures and models in the primary study are marginal in significance, possibly due to study size and resulting borderline power; the finding of a significant association in the replication study provides additional evidence for the rs876538 association. The lack of replication seen in the European ancestry samples for rs1205 and rs3093068 may be due to differences in LD, or unidentified phenotypic or environmental factors differing between populations.

Limitations of this study include limited power due to modest sample size which is common to many investigations of PE, although we emphasize that this is the largest PE study to date in American Indians. Our multivariate logistic regression results nearly reached study-wide significance after conservative, Bonferroni adjustment for multiple testing only for the additive risk score among severe cases. However, our previous finding of association with rs1205 in this cohort, replication of results for rs876538 in another population, and nominally significant findings in two of ten tested SNPs, certainly suggests that the *CRP* gene warrants further investigation. The fact that 24% of our controls were recruited in a slightly different fashion and the difference between case and control delivery dates was slightly over 9 days greater than the other case/control pairs could have possibly introduced a subtle, unrecognized bias, although concern for seasonality effects of this degree seems unwarranted. The inability to adjust the replication sample for clinical covariates was also a limitation but we again emphasize that this is a unique study population. Strengths of this study include a well-defined phenotype of PE and the study design which employed an unbiased ascertainment of cases and controls. The fact that the present study focused on *CRP* gene variants rather than circulating CRP levels, should reduce the extraneous effects of multiple environmental factors on *CRP* expression.

Our findings related to these 2 previously unreported *CRP* gene variants provide suggestive findings that may warrant further expression studies and replication in other cohorts. Although there is a significant correlation between these SNPs, the combined strength of association for both rs3093068 and rs876538 with severe PE risk were enhanced, compared with either assessed separately suggesting that these gene variants do not merely share a haplotype. Until our publication reporting the significant association of the CRP SNP rs1205[31], associations of CRP with PE were limited to the circulating protein product. In this study, we extended our investigation reporting two additional SNPs, rs3093068 and rs876538, likely associated with PE in an American Indian population and evidence for a similar association of rs876538 in those of European ethnicity. Our findings, combined with clinical correlates, may contribute the prioritization of biological processes underpinning this devastating disease in women at risk for developing PE.

## Supporting Information

Figure S1 **Genomic context of CRP, Chromosome 1 extended locus.** Data is visualized using the UCSC human genome browser and custom track data, presented in the following order: i) AMR LD proxy SNPs (r2 LD>0.5), ii) ASN LD proxy SNPs (r2 LD>0.5), iii) CEU LD proxy SNPs (r2 LD>0.5), iv) Directly associated SNPs, v) Liver EST sequence BG616599, vi) Published studies of CRP including downstream regions of functional characterization, vi) ENCODE: RNA-seq assayed in 9 cell lines, vii) ENCODE: H3K4Me3 histone marks representing probable promoter activity, viii) ENCODE: H3KMe1 histone marks presenting probable regulatory enhancer activity, ix) ENCODE: H3K27Ac histone marks presenting probable regulatory enhancer activity, x)ENCODE: DNase I hypersensitive regions, indicating DNA binding activity, xi) Liver tissue RNA-seq from the Burge lab, xii) Mammalian conservation.
(TIF)

Figure S2 **Chromosome 1 directly associated SNPs.** Data is visualized using the UCSC human genome browser and custom track data, presented in the following order: i) AMR LD proxy SNPs (r2 LD>0.5), ii) ASN LD proxy SNPs (r2 LD>0.5), iii) CEU LD proxy SNPs (r2 LD>0.5), iv) Directly associated SNPs, v) Liver EST sequence BG616599, vi) Published studies of CRP including downstream regions of functional characterization, vi) ENCODE: RNA-seq assayed in 9 cell lines, vii) ENCODE: H3K4Me3 histone marks representing probable promoter activity, viii) ENCODE: H3KMe1 histone marks presenting probable regulatory enhancer activity, ix) ENCODE: H3K27Ac histone marks presenting probable regulatory enhancer activity, x)ENCODE: DNase I hypersensitive regions, indicating DNA binding activity, xi) Liver tissue RNA-seq from the Burge lab, xii) Mammalian conservation.
(TIF)

Table S1 **Further detail concerning SNPs shown in Figure S2.**
(XLS)
