# Correction: Two Variants of the C-Reactive Protein Gene Are Associated with Risk of Pre-Eclampsia in an American Indian Population

**DOI:** 10.1371/annotation/7f3e30b8-8dfa-4bb7-b34e-884248628454

**Published:** 2013-09-23

**Authors:** Lyle G. Best, Richa Saxena, Cindy M. Anderson, Michael R. Barnes, Hakon Hakonarson, Gilbert Falcon, Candelaria Martin, Berta Almoguera Castillo, Ananth Karumanchi, Kylie Keplin, Nichole Pearson, Felicia Lamb, Shellee Bercier, Brendan J. Keating

There was information omitted from the Competing Interests statement.

The information is: "Dr. Karumanchi is a co-inventor on patents related to preeclampsia diagnosis/therapy that is held by Beth Israel Deaconess Medical Center. Dr. Karumanchi also reports having served as a consultant to Beckman Coulter, Siemens, and Roche and has financial interest in Aggamin LLC." 

