# Supplementary figures and images for: Two Variants of the C-Reactive Protein Gene Are Associated with Risk of Pre-Eclampsia in an American Indian Population
Source: PLoS One. 2013 Aug 5;8(8):e71231. doi: 10.1371/journal.pone.0071231 (PMC3733916; doi:10.1371/journal.pone.0071231)

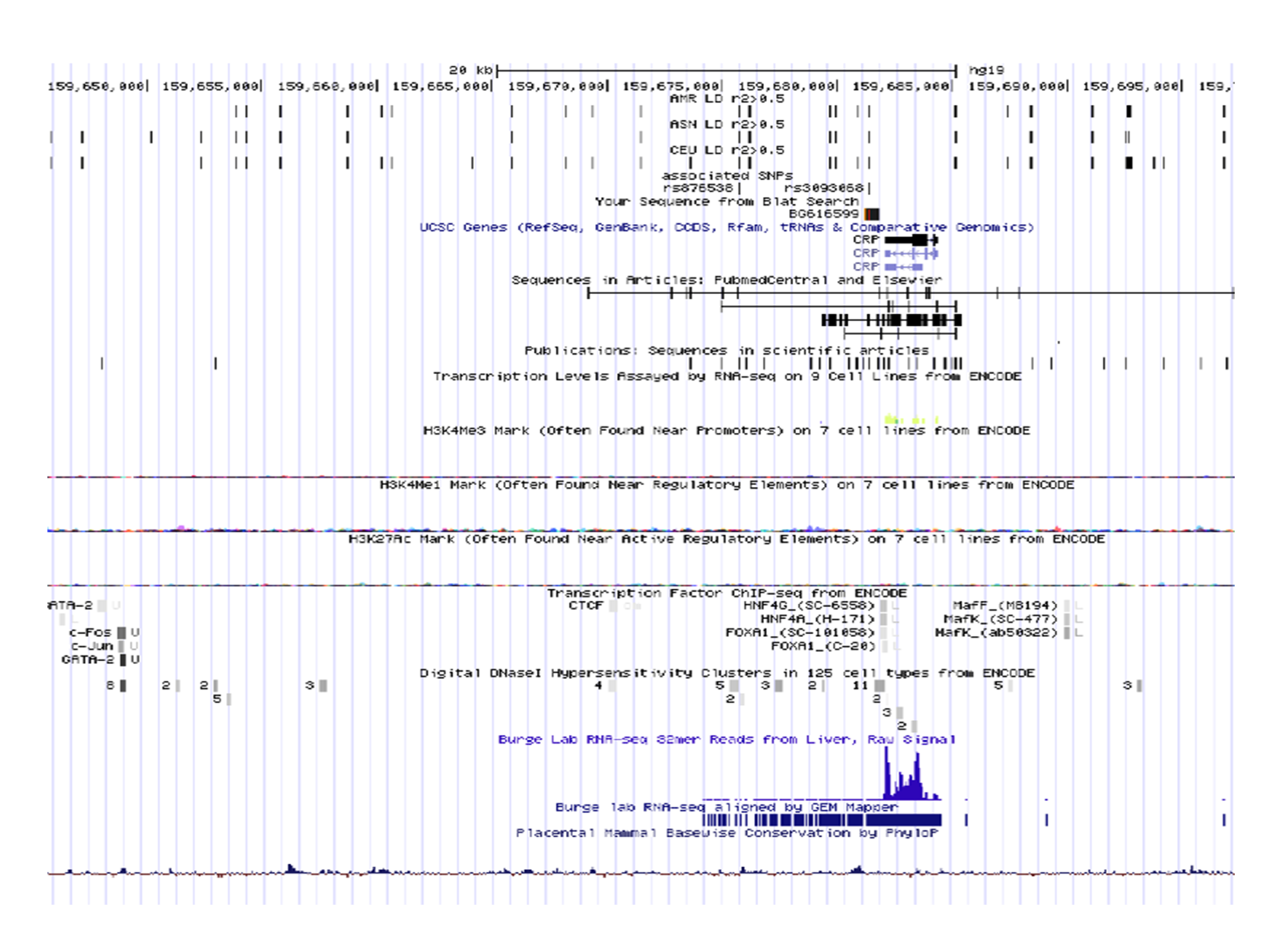

Supplement: Figure S1 — Genomic context of CRP, Chromosome 1 extended locus. Data is visualized using the UCSC human genome browser and custom track data, presented in the following order: i) AMR LD proxy SNPs (r2 LD>0.5), ii) ASN LD proxy SNPs (r2 LD>0.5), iii) CEU LD proxy SNPs (r2 LD>0.5), iv) Directly associated SNPs, v) Liver EST sequence BG616599, vi) Published studies of CRP including downstream regions of functional characterization, vi) ENCODE: RNA-seq assayed in 9 cell lines, vii) ENCODE: H3K4Me3 histone marks representing probable promoter activity, viii) ENCODE: H3KMe1 histone marks presenting probable regulatory enhancer activity, ix) ENCODE: H3K27Ac histone marks presenting probable regulatory enhancer activity, x)ENCODE: DNase I hypersensitive regions, indicating DNA binding activity, xi) Liver tissue RNA-seq from the Burge lab, xii) Mammalian conservation. (TIF) [file pone.0071231.s001.tif]

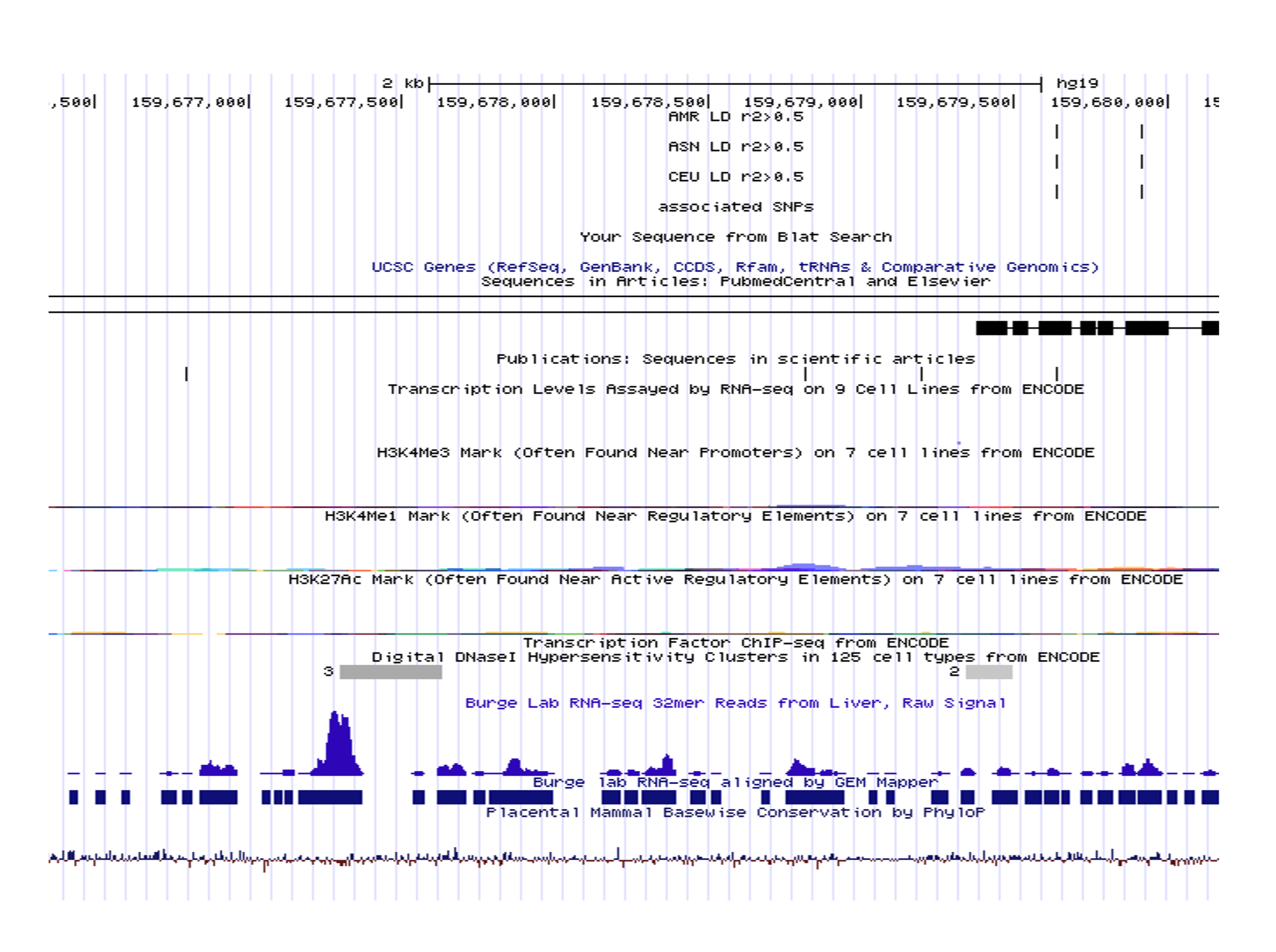

Supplement: Figure S2 — Chromosome 1 directly associated SNPs. Data is visualized using the UCSC human genome browser and custom track data, presented in the following order: i) AMR LD proxy SNPs (r2 LD>0.5), ii) ASN LD proxy SNPs (r2 LD>0.5), iii) CEU LD proxy SNPs (r2 LD>0.5), iv) Directly associated SNPs, v) Liver EST sequence BG616599, vi) Published studies of CRP including downstream regions of functional characterization, vi) ENCODE: RNA-seq assayed in 9 cell lines, vii) ENCODE: H3K4Me3 histone marks representing probable promoter activity, viii) ENCODE: H3KMe1 histone marks presenting probable regulatory enhancer activity, ix) ENCODE: H3K27Ac histone marks presenting probable regulatory enhancer activity, x)ENCODE: DNase I hypersensitive regions, indicating DNA binding activity, xi) Liver tissue RNA-seq from the Burge lab, xii) Mammalian conservation. (TIF) [file pone.0071231.s002.tif]
